# Clinical significance and oncogenic role of ECHDC2 in glioblastoma: a comprehensive analysis based on bioinformatics and *in vitro* experiments

**DOI:** 10.3389/fgene.2026.1759463

**Published:** 2026-02-09

**Authors:** Shengliang Lin, Tian Wei, Qian Wu, Qingqing Liu, Longyun Hu, Bigui Song, Jiejing Lin, Zewei Zhao, Yi Cai, Xiaoxiao Li, Zhonghan Yang, Chengming Li, Xiping Hu

**Affiliations:** 1 School of Medical Technology, Beijing Institute of Technology, Beijing, China; 2 Artificial Intelligence Research Institute, Shenzhen MSU-BIT University, Shenzhen, Guangdong, China; 3 Shenzhen Key Laboratory of Systems Medicine for inflammatory diseases, School of Medicine, Shenzhen Campus of Sun Yat-Sen University, Sun Yat-Sen University, Shenzhen, Guangdong, China; 4 Department of Biochemistry, Zhongshan School of Medicine, Sun Yat-Sen University, Guangzhou, China

**Keywords:** bioinformatics, ECHDC2, glioblastoma, immune infiltration, PI3K/AKT

## Abstract

**Background:**

Growing evidence implicates enoyl-CoA hydratase domain-containing protein 2 (ECHDC2) in oncogenesis, yet its role in glioblastoma (GBM) remains undefined. We aimed to clarify the pathological significance and molecular mechanisms of ECHDC2 in GBM.

**Methods:**

Gene-expression profiles from The Cancer Genome Atlas (TCGA) and Gene Expression Omnibus (GEO) databases were analyzed. Kaplan–Meier curves were used to evaluate the prognostic value of ECHDC2. Immune cell infiltration was quantified using CIBERSORT, single-sample gene-set enrichment analysis (ssGSEA), and ESTIMATE algorithms. Spearman’s correlation analysis was applied to assess the associations between ECHDC2 expression levels, immune checkpoint molecules, and immune cell subsets. To elucidate the functional relevance of ECHDC2, Gene Ontology (GO), Kyoto Encyclopedia of Genes and Genomes (KEGG), and gene-set enrichment analyses (GSEA) were performed, while protein-protein interaction (PPI) networks were investigated using the STRING database. Subsequently, ECHDC2 was knocked down or overexpressed in GBM cell lines, and its effects on cell proliferation and migration were determined using CCK-8, EdU, wound-healing, and Transwell migration assays.

**Results:**

Upregulated ECHDC2 expression was significantly correlated with unfavorable clinicopathological features and reduced overall survival (OS) in patients with GBM. High ECHDC2 expression was associated with increased infiltration of effector-memory CD8^+^ T cells (TEM) and plasmacytoid dendritic cells (pDCs). Enrichment analyses demonstrated that ECHDC2 is involved in tumor progression, with a particular focus on the PI3K/Akt signaling pathway. *In vitro* experiments showed that ECHDC2 knockdown suppressed the proliferation and migration of GBM cells. Conversely, ECHDC2 overexpression exerted the opposite effects on GBM cell proliferation and migration.

**Conclusion:**

ECHDC2 overexpression promotes GBM progression and portends poor prognosis. ECHDC2 may serve as both a prognostic biomarker and a therapeutic target in GBM.

## 1 Introduction

Glioblastoma (GBM) is the most malignant primary brain tumor in adults and carries the bleakest prognosis. Although surgical resection followed by radiotherapy and/or chemotherapy remains the therapeutic mainstay, overall survival is dismal: fewer than 25% of patients are alive 1 year after diagnosis (Stupp et al., 2005; Wen and Kesari, 2008; Ostrom et al., 2019; Jiang et al., 2022). In addition to conventional prognostic factors—tumor size, stage and circulating tumor cells—patient outcome is heavily influenced by the dynamic interplay between malignant cells and the tumor micro-environment, which orchestrates tumor initiation, progression, metastasis and therapeutic response (Xiao and Yu, 2021; Yu et al., 2024). Identifying reliable molecular markers that can refine prognosis and inform treatment decisions is therefore an urgent clinical priority.

Metabolic reprogramming is integral to tumor cell proliferation, invasion and metastasis (Currie et al., 2013; Li and Zhang, 2016). In particular, heightened *de novo* lipogenesis is a hallmark metabolic aberration that fuels oncogenesis (Swierczynski et al., 2014). Cancer cells depend on fatty acids (FAs) for membrane biogenesis, energy storage and the synthesis of signalling molecules. Mitochondrial FA β-oxidation catabolises FAs into acetyl-CoA, generating adenosine triphosphate (ATP), NADH and FADH_2_ to satisfy cellular energy requirements (Carracedo et al., 2013; Koundouros and Poulogiannis, 2020). In GBM, upregulated FA metabolism facilitates immune evasion and drives aggressive growth (Jiang et al., 2022). The enoyl-CoA hydratase/isomerase family is indispensable for FA metabolism, coupling β-oxidation to other metabolic pathways, including aerobic glycolysis, thereby supporting tumor growth and adaptability (Padavattan et al., 2021; Agnihotri and Liu, 2003). Enoyl-CoA-hydratase-domain-containing protein 2 (ECHDC2) has been shown to suppress proliferation and aerobic glycolysis in gastric cancer cells (He et al., 2024), but its function in GBM remains unexplored.

Here, we characterize the expression profile and biological role of ECHDC2 in GBM and assess its utility as a prognostic biomarker and potential therapeutic target.

## 2 Materials and methods

### 2.1 Data collection and preprocessing

Transcriptomic profiles and matched clinical information for GBM were retrieved from The Cancer Genome Atlas (TCGA; https://portal.gdc.cancer.gov), the Gene Expression Omnibus (GEO; https://www.ncbi.nlm.nih.gov/geo), the Chinese Glioma Genome Atlas (CGGA; http://www.cgga.org.cn) and the Ivy Glioblastoma Atlas Project (Ivy GAP; https://glioblastoma.alleninstitute.org). Bulk-RNA-seq datasets (TCGA-GBM, CGGA-325, CGGA-693, GSE43378, GSE13041, GSE74187 and GSE83300) and a single-cell RNA-sequencing (scRNA-seq) dataset (GSE182109) were downloaded. Ensembl gene identifiers were converted to gene symbols, clinical variables were matched to each sample, and all datasets were merged in R (v 4.1.2). ECHDC2 was identified by intersecting genes with significant prognostic value in Kaplan–Meier analysis across the GSE83300, GSE43378, GSE74187, and GSE13041 datasets, followed by subsequent analyses (Supplementary Figure S1).

### 2.2 Single-cell RNA-seq analysis

scRNA-seq data were processed with Seurat package (v 4.3.0.1) (Hao et al., 2021). Quality-control filters were applied to retain cells with 200 < nFeature_RNA <4 000, nCount_RNA <30 000 and percent. mt < 20%. Batch effects were corrected with Harmony package (v 0.1.1). Neighbour graphs and clusters were generated with FindNeighbors and FindClusters, respectively, and visualised by Uniform Manifold Approximation and Projection (UMAP). Cell types were annotated on the basis of canonical marker genes.

### 2.3 Diagnostic and prognostic evaluation

Time-dependent receiver-operating-characteristic (ROC) curves were generated with “timeROC” package (v 0.4). Patients were stratified into high- and low-ECHDC2 groups according to the median ECHDC2 mRNA level. Overall survival was compared by Kaplan–Meier analysis using “survival” package (v 3.7–0).

### 2.4 Immune-infiltration analysis

Relative immune-cell infiltration was estimated by single-sample gene-set enrichment analysis (ssGSEA) implemented in “GSVA” package (v 1.48.3) (Hänzelmann et al., 2013). Absolute immune-cell fractions were inferred with CIBERSORT (Newman et al., 2015), which distinguishes 22 immune-cell subsets on the basis of 547 marker genes. Stromal, immune and ESTIMATE scores of the tumor micro-environment (TME) were calculated with “ESTIMATE” package (v 1.0.13) (Yoshihara et al., 2013). Correlations between ECHDC2 expression and cytokines, chemokines or immune checkpoints were evaluated by Spearman’s test and Wilcoxon rank-sum test (two-tailed; p < 0.05). Intersections across datasets were depicted with Venn diagrams.

### 2.5 Protein-interaction and functional-enrichment analyses

A protein-protein interaction (PPI) network centered on ECHDC2 was constructed using the STRING database (Szklarczyk et al., 2021) (http://www.stringdb.org/). Based on the CGGA-325 RNA-seq dataset, we identified differentially expressed genes (DEGs) between high and low ECHDC2 expression groups using the limma package (v 3.56.2) in R (v 4.1.2), with the median expression level of ECHDC2 as the cutoff. The specific screening criteria were: |log2FoldChange| > 1 and adjusted P-value < 0.05. The upregulated and downregulated DEGs were merged into a single gene set for subsequent analyses (Supplementary Table S1). Gene Ontology (GO) enrichment analysis, Kyoto Encyclopedia of Genes and Genomes (KEGG) enrichment analysis, and Gene Set Enrichment Analysis (GSEA) were performed using the “clusterProfiler” package (v 4.8.3) (Wu et al., 2021).

### 2.6 Pan-cancer analysis

ECHDC2 mRNA expression across cancer types was obtained from the Cancer Cell Line Encyclopedia (CCLE) (https://sites.broadinstitute.org/ccle/) and the TIMER web server (https://cistrome.shinyapps.io/timer) (Li T. et al., 2017). Normal- and tumor-tissue RNA-seq data were downloaded from TCGA. Kaplan-Meier curves and univariate Cox regression were generated with “survival” package (v 3.7–0) and visualised with “survminer” package (v 0.5.0) to evaluate overall survival (OS), disease-specific survival (DSS), disease-free interval (DFI) and progression-free interval (PFI).

### 2.7 Cell culture and transfection

Human GBM U251 and A172 cells (Cell Bank, Chinese Academy of Sciences, Shanghai, China) were cultured in DMEM (C119955008BT, Gibco) supplemented with 10% fetal bovine serum (A5256701, Gibco) at 37 °C in a humidified 5% CO_2_ incubator. Three small interfering RNAs (siRNAs) targeting ECHDC2 (si-ECHDC2-1: 5′-GGC​CGA​CGA​CUG​AGU​GGA​A-3′; si-ECHDC2-2: 5′-GGA​AUG​UCU​UCG​UCA​GUG​A-3′; si-ECHDC2-3: 5′-CGU​GUU​CUG​UGC​AGG​UGC​A-3′) were synthesized by OBiO (Shanghai, China). Transfections were performed with Lipofectamine 3000 (L3000-015, Invitrogen) in Opti-MEM (31985070, Gibco).

### 2.8 Quantitative real-time polymerase chain reaction (qRT-PCR)

Total RNA was extracted with the SteadyPure Rapid RNA kit (AG21023, Accurate Biology) and reverse-transcribed using EvoScript RT premix (AG11706, Accurate Biology). The qRT-PCR was performed with a SYBR Green Pro Taq HS kit (AG11718, Accurate Biology). Relative expression was calculated by the 2^-ΔΔCT^ method, normalizing to β-actin. The sequences of primers were as follows: ECHDC2, Forward primer 5′-AGT​GCG​TGT​CCT​GCT​CTT​C-3′, Reverse primer 5′-CAT​CTG​TTC​CCG​CTC​CTT​CA-3’; β-actin, Forward primer 5′-CCT​TTG​CCG​ATC​CGC​CG-3′, Reverse primer 5′-AAT​CCT​TCT​GAC​CCA​TGC​CC-3’. AKT, Forward primer 5′-CTG​CAC​AAA​CGA​GGG​GAG​TA-3′, Reverse primer 5′-GCG​CCA​CAG​AGA​AGT​TGT​TG-3’.

### 2.9 Western blotting

Cells were lysed in SDS buffer containing protease and phosphatase inhibitors (HY-K0010 and HY-K0021, MCE). Proteins were separated on 10% SDS-PAGE gels and transferred to PVDF membranes (IPVH00010, Merck). After blocking (PS108P, Yamei), membranes were incubated overnight at 4 °C with anti-ECHDC2 (26126-1-AP, Proteintech; 1:1000), phosphorylated AKT (p-AKT, Ser473 phosphorylation) antibody (AF0016, Affinity; 1:1000), AKT antibody (AF6261, Affinity; 1:1000), or anti-β-actin (4970S, Cell Signaling Technology; 1:2000), followed by HRP-conjugated secondary antibody (7074S, Cell Signaling Technology; 1:2000) and chemiluminescence detection.

### 2.10 Cell counting kit-8 assays

The CCK-8 Cell Counting Kit (C6005, NCM Biotech) was used to measure the proliferation ability of GBM cells. For CCK-8 assays, 100 ul of medium containing 1*10^3^ cells was added to each well of a 96-well plate. Before measuring, the medium in the wells to be tested was replaced with fresh medium containing 10% CCK-8, and then placed in an incubator for 2 h. Then a microplate reader was used to measure the OD of the wells to be tested at a wavelength of 450 nm.

### 2.11 EdU assay

Edu assay was performed using Edu kit (C0075S, Beyotime), according to the instructions. The EdU solution was prepared as a 1:1000 concentration of medium and added to the pre-prepared 12-well plates with 500 ul per well, which were incubated at 37 °C for 2 h and fixed in 4% paraformaldehyde for 15 min. The fixed cells were incubated with 100 ul of reaction solution for 30 min sheltered from light, and then the nuclei were stained for 10 min by using DAPI. Cell proliferation was imaged by utilization of a 20x fluorescence microscope.

### 2.12 Transwell migration assays

Cell migration and invasion assays were performed using 6.5 mm Transwell chambers with 8.0 μm pore polycarbonate membrane inserts (3422, Corning). For the migration assay, 650 μL of medium containing 10% FBS was added to the lower chamber, and then 200 μL of serum-free cell suspension (2*10^4^ cells) was seeded into the upper chamber. The cells were incubated at 37 °C for 24 h, followed by fixation with 4% paraformaldehyde and staining with crystal violet solution. The stained cells in the upper chamber were gently removed, and the cells that migrated to the underside of the membrane were imaged and counted under a light microscope.

For the invasion assay, before seeding the cells (4*10^4^ cells) into the upper chamber, the membrane of the upper chamber was coated with 60 μL of Matrigel (diluted 1:8, 354234, Corning). The remaining steps were identical to those of the migration assay.

### 2.13 Wound-healing assay

Confluent monolayers were scratched with a sterile 200 µL pipette tip held perpendicular to the plate. After washing away debris, cells were incubated in 2% FBS medium, and wound closure was photographed at 0 h and 24 h. Migratory distance was quantified in ImageJ (v 1.53q).

### 2.14 Statistical analysis

Data are presented as mean ± standard deviation. Two-group comparisons were performed with unpaired two-tailed Student’s t-tests; multiple-group comparisons used one-way ANOVA followed by Tukey’s post hoc test. p < 0.05 was considered statistically significant. Statistical analyses were conducted in R (v 4.1.2) or GraphPad Prism (v 9.0.0).

## 3 Results

### 3.1 ECHDC2 is upregulated in high-grade glioma

**FIGURE 1:**
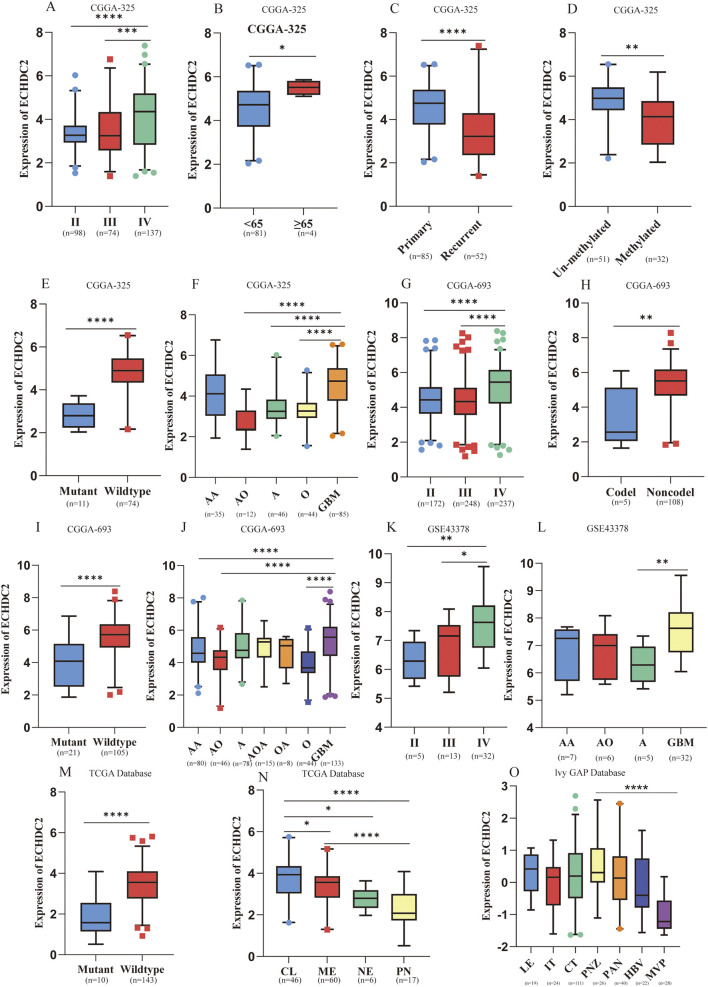
Relationship between ECHDC2 expression and clinicopathological characteristics in GBM. **(A–F)** Distribution of ECHDC2-associated clinical features in GBM patients from the CGGA-325 dataset. **(G–J)** Distribution of ECHDC2-associated clinical features in GBM patients from the CGGA-693 dataset. **(K,L)** Association between ECHDC2 expression and World Health Organization (WHO) grade as well as pathological subtype in the GSE43378 dataset. **(M,N)** Association of ECHDC2 expression with IDH mutation status and TCGA molecular subtype in the TCGA cohort. **(O)** Spatial expression pattern of ECHDC2 across GBM histologic structures in the Ivy GAP database. Abbreviations: NE, neural; PN, proneural; ME, mesenchymal; CL, classical; A, astrocytoma; AA, anaplastic astrocytoma; GBM, glioblastoma; OA, oligoastrocytoma; O, oligodendroglioma; AO, anaplastic oligodendroglioma; AOA, anaplastic oligoastrocytoma. Significance: ns: no significance; *P < 0.05; **P < 0.01; ***P < 0.001; ****P < 0.0001.

**FIGURE 2:**
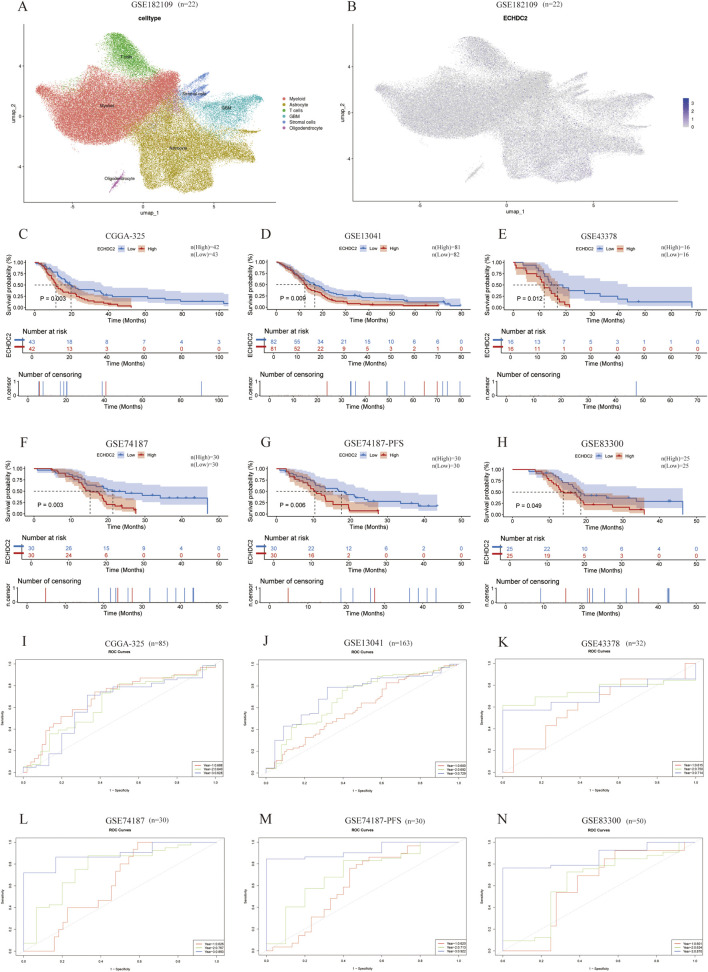
Single-cell expression atlas of ECHDC2 and its prognostic significance in GBM. **(A)** UMAP plot illustrating the distribution of GBM single-cell clusters annotated by cell type. **(B)** ECHDC2 expression across the identified cell types. **(C–E,H)** Kaplan–Meier OS curves stratified by high versus low ECHDC2 expression in the CGGA-325, GSE13041, GSE43378 and GSE83300 cohorts, respectively. **(F,G)** Kaplan-Meier OS and PFS curves comparing high and low ECHDC2 expression in the GSE74187 cohort. **(I–N)** Time-dependent ROC analyses evaluating the prognostic accuracy of ECHDC2 in the CGGA-325, GSE13041, GSE43378, GSE74187 and GSE83300 cohorts.

Across CGGA, GEO, TCGA and Ivy GAP cohorts, ECHDC2 mRNA was markedly elevated in WHO grade III and grade IV gliomas (Figures 1A,G,K). Higher ECHDC2 expression correlated with older age (Figure 1B), primary rather than recurrent tumors (Figure 1C), an unmethylated MGMT promoter (Figure 1D) and 1p/19q non-codeletion (Figure 1H). Expression was significantly greater in IDH-wild-type GBM than in IDH-mutant tumors (Figures 1E,I,M) and highest in GBM relative to lower-grade gliomas (Figures 1F,J,L). Within molecular or anatomical subclasses, ECHDC2 was enriched in the TCGA Classical subtype (Figure 1N) and in pseudopalisading necrosis zones (PNZ; Figure 1O). These observations indicate that ECHDC2 expression increases with tumor grade and is prevalent in key GBM niches. Notably, Single-cell RNA-seq data (GSE182109) identified six principal cell clusters (Figure 2A); ECHDC2 was broadly expressed in malignant cells, astrocytes and myeloid cells (Figure 2B).

### 3.2 High ECHDC2 expression predicts poor prognosis in GBM

Patients in CGGA-325, GSE13041, GSE43378, GSE74187 and GSE83300 were stratified by the median ECHDC2 level. Kaplan-Meier analysis demonstrated that high ECHDC2 expression conferred shorter OS in CGGA-325 (p = 0.003; Figure 2C) and in the four GEO cohorts (p = 0.009–0.049; Figures 2D–F,H). Progression-free survival (PFS) was likewise reduced (p = 0.006; Figure 2G). Time-dependent ROC curves yielded area-under-the-curve (AUC) values of 0.688, 0.640 and 0.628 at 1, 2 and 3 years in CGGA-325 (Figure 2I), with comparable AUCs (0.600–0.922) in GEO datasets (Figures 2J–N). Thus, elevated ECHDC2 is a robust adverse prognostic marker in GBM.

### 3.3 Immune landscape and pathway enrichment associated with ECHDC2

**FIGURE 3:**
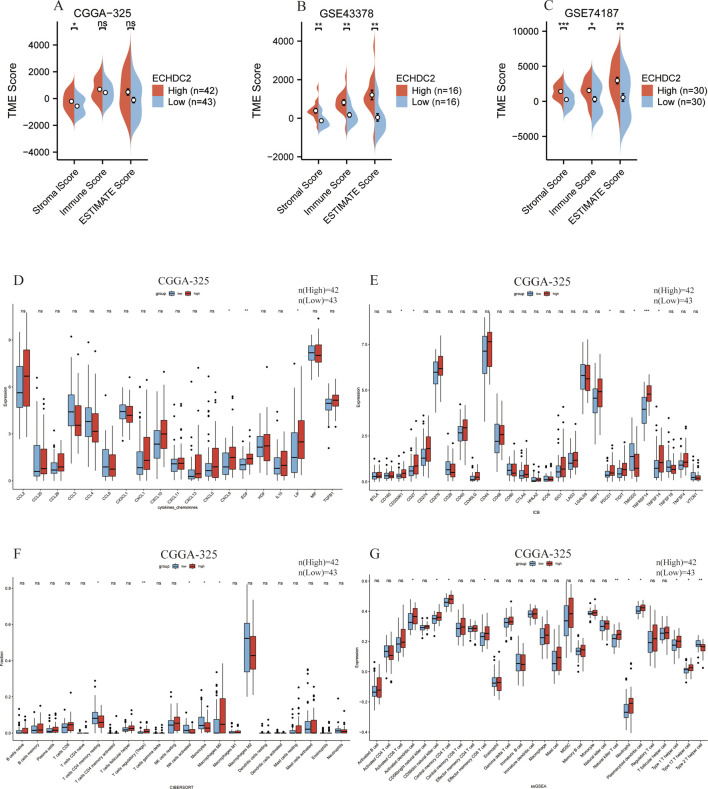
ESTIMATE analysis and immune-infiltration landscape associated with ECHDC2 expression in GBM. **(A–C)** Pod plots comparing Stromal Score, Immune Score and ESTIMATES core between the high- and low-ECHDC2 expression groups as calculated by the ESTIMATE algorithm. **(D–G)** Differential expression of cytokine, chemokine, immune checkpoint blockade genes CIBERSORT and ssGSEA between ECHDC2-high and -low groups in the CGGA-325 cohorts. Significance: ns: no significance; *P < 0.05; **P < 0.01; ***P < 0.001; ****P < 0.0001.

Across five bulk RNA-seq datasets, Stromal, Immune, and ESTIMATE scores were each positively correlated with ECHDC2 expression (Figures 3A–C; Supplementary Figures 2A, B). These findings indicate that patients with high ECHDC2 expression exhibit a highly active TME, increased immune infiltration, and thus may be more sensitive to immunotherapy. Accordingly, we stratified analyses of cytokines (Figure 3D; Supplementary Figures 2C–F), immune checkpoints (Figure 3E; Supplementary Figures 2G–J), and infiltrating immune cells by ECHDC2 expression levels (Figures 3F,G; Supplementary Figures 2K–R).

**FIGURE 4:**
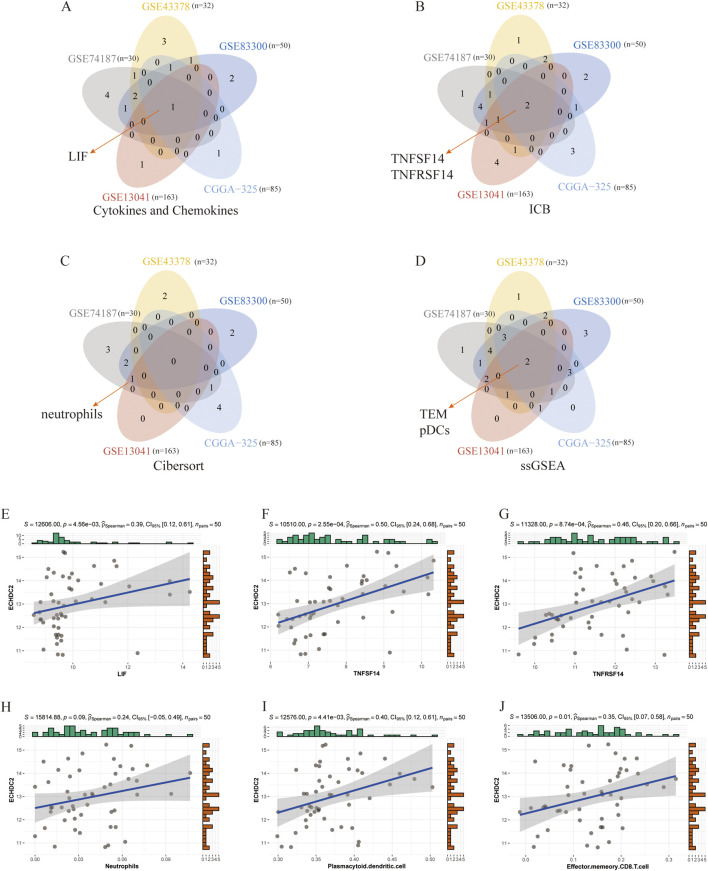
Associations between ECHDC2 expression and immunological features in the GSE83300 cohort. **(A–D)** Venn diagrams highlighting the cytokines, chemokines, immune-checkpoint genes, and immune-cell populations commonly altered in the above datasets. **(E)** Scatter plot illustrating the correlation between ECHDC2 transcript abundance and the cytokine LIF. **(F)** Scatter plots showing the relationships between ECHDC2 expression and TNFSF14. **(G)** Scatter plots showing the relationships between ECHDC2 expression and TNFRSF14. **(H–J)** Correlations between ECHDC2 expression and neutrophils, plasmacytoid dendritic cell and effector memory CD8^+^ T cells. Abbreviations: LIF, leukemia inhibitory factor; TEM, effector-memory CD8^+^ T cells; pDCs, plasmacytoid dendritic cells. Significance: ns: no significance; *P < 0.05; **P < 0.01; ***P < 0.001; ****P < 0.0001.

Across the five bulk datasets, ECHDC2 expression was positively correlated with the cytokine leukemia inhibitory factor (LIF) (Figures 4A,E) and the immune checkpoint pair TNFSF14/TNFRSF14 (Figures 4B,F,G), suggesting an immunomodulatory role for ECHDC2. CIBERSORT analysis identified neutrophils as the most frequently intersecting immune cell population among 22 immune subsets (Figure 4C), although neutrophil abundance was not significantly correlated with ECHDC2 expression (Figure 4H). ssGSEA results revealed higher enrichment levels of effector-memory CD8^+^ T cells (TEM) and plasmacytoid dendritic cells (pDCs) in tumors with high ECHDC2 expression (Figure 4D), and both of these immune cell populations were positively correlated with ECHDC2 (Figures 4I,J).

**FIGURE 5:**
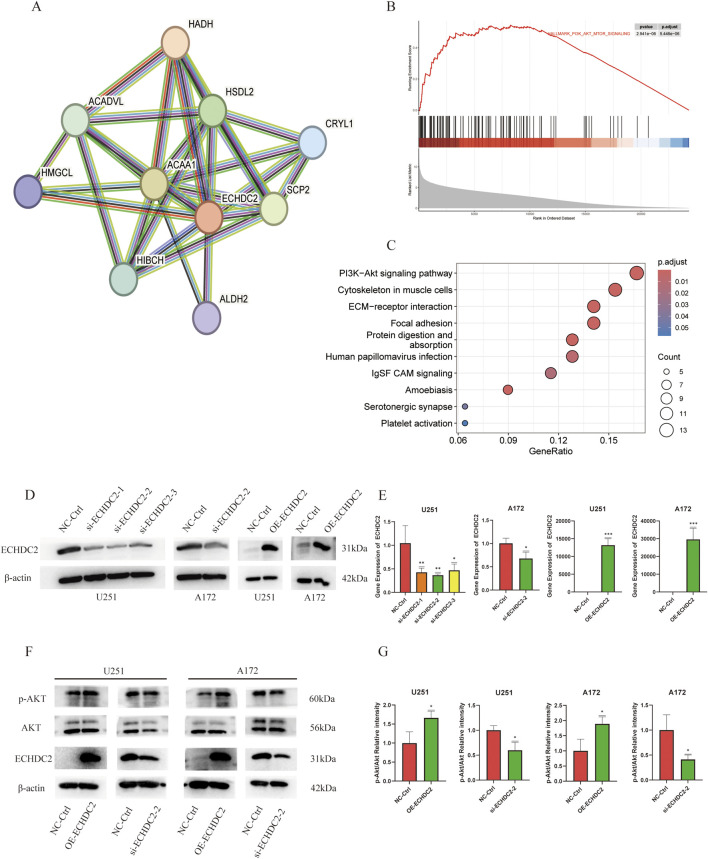
Functional enrichment analyses and impact of ECHDC2 on the PI3K/Akt signaling pathway. **(A)** STRING-derived PPI network illustrating the interactions between ECHDC2 and its top predicted interactors. **(B)** GSEA demonstrating significant positive enrichment of the PI3K/Akt pathway in tumors with high ECHDC2 expression in CGGA-325 cohort. **(C)** KEGG pathway enrichment analyses for the ECHDC2-high and -low groups in CGGA-325 cohort. **(D,E)** The efficiency of ECHDC2 knockdown and overexpression was confirmed by immunoblotting and qPCR analysis, respectively. **(F,G)** Western blot analyses of p-AKT (Ser473 phosphorylation) and AKT in GBM cells with ECHDC2 overexpression or knockdown.

A protein–protein interaction (PPI) network constructed using STRING localized ECHDC2 to a metabolic hub, with ACAA1, HADH, HSDL2, SCP2, and HMGCL as its core interacting partners (Figure 5A). KEGG and GSEA enrichment analyses indicated that ECHDC2 may contribute to the promotion of GBM progression via the PI3K/Akt signaling pathway (Figures 5B,C). GO enrichment analyses emphasized the potential involvement of ECHDC2 in the biological processes of gliogenesis, glial cell differentiation, and glial cell development (Supplementary Figure S3A).

### 3.4 ECHDC2 knockdown suppresses GBM cell proliferation and migration

**FIGURE 6:**
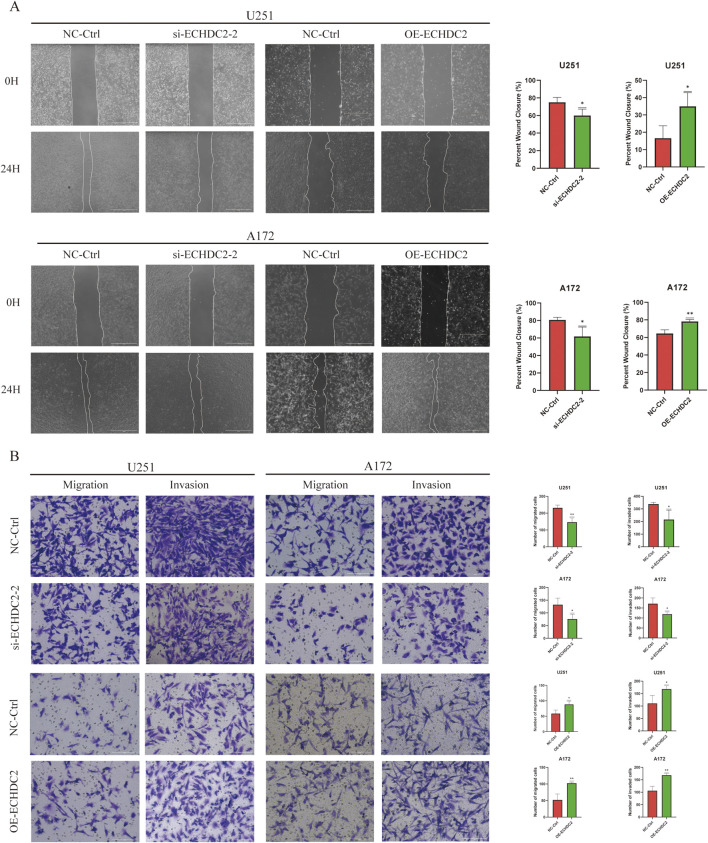
ECHDC2 promotes GBM cell migration. **(A)** Wound healing assays were performed to assess the migration of U251 and A172 cells following ECHDC2 knockdown or overexpression, compared to control cells. **(B)** Cell migration and invasion were evaluated in U251 and A172 cells with ECHDC2 knockdown or overexpression using Transwell assays, relative to controls.

**FIGURE 7:**
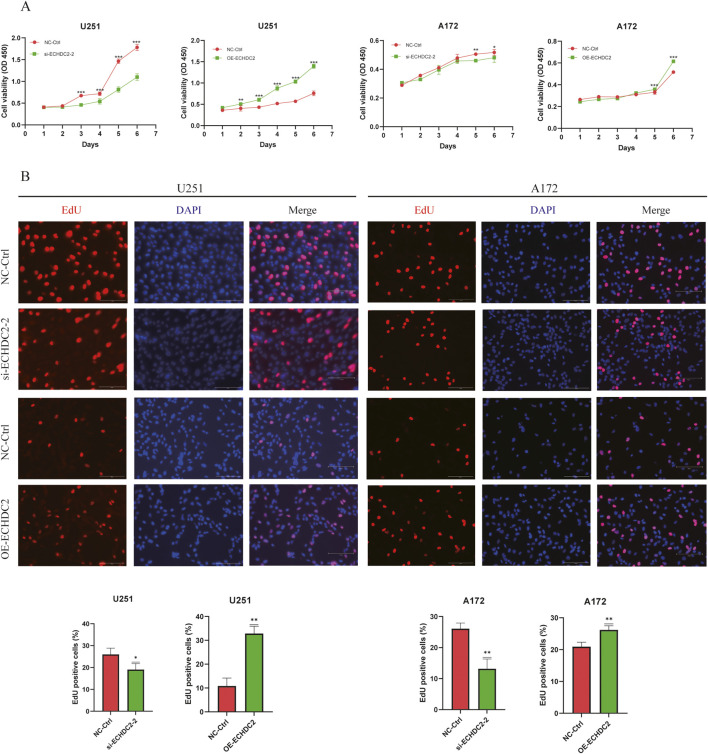
ECHDC2 promotes GBM cell proliferation. **(A)** Cell proliferation was assessed in U251 and A172 cells following ECHDC2 knockdown or overexpression, relative to controls. **(B)** Proliferation of U251 and A172 cells with ECHDC2 knockdown or overexpression was evaluated by EdU staining, compared to control cells.

To elucidate the functional role of ECHDC2 in GBM cells, we knocked down or overexpressed ECHDC2 in GBM cells by transfecting ECHDC2-targeting siRNAs or ECHDC2-overexpressing plasmids, respectively. The knockdown and overexpression efficiencies of ECHDC2 were detected via Western blotting and qRT-PCR, respectively, in both U251 and A172 cells (Figures 5D,E). The ECHDC2-targeting siRNA #2, which exhibited the highest knockdown efficiency, was selected for subsequent functional experiments. The level of p-AKT was significantly decreased in ECHDC2-knockdown cells, while it was increased in ECHDC2-overexpressing cells, with no obvious alterations in total AKT expression (Figures 5F,G; Supplementary Figure S3B). Compared with cells in the negative control (NC) group, GBM cells with ECHDC2 knockdown showed significantly inhibited migration and invasion capacities, whereas ECHDC2-overexpressing cells exhibited the opposite effects. These findings were validated by both wound-healing and Transwell assays (Figures 6A,B). Furthermore, ECHDC2 knockdown suppressed the proliferative capacity of GBM cells, while ECHDC2 overexpression exerted the opposite effect (Figure 7A). Consistent results were obtained in the EdU assay (Figure 7B).

### 3.5 Pan-cancer expression, survival impact and immune associations

CCLE data indicated ubiquitous ECHDC2 transcription across normal tissues (Supplementary Figure S4A). TCGA analysis showed reduced ECHDC2 in 14 cancers—including BRCA, CHOL and COAD—and increased expression in PRAD (Supplementary Figure S4B).

Univariate Cox regression across 32 tumors linked ECHDC2 to OS and DSS in nine cancers (Supplementary Figure S4C). ECHDC2 acted as a high-risk gene in THYM and LGG, but as a protective factor in bladder, head-and-neck, kidney, liver, lung, mesothelioma and pancreatic cancers. Kaplan–Meier curves mirrored these trends (Supplementary Figures S4F–N). For DFI, ECHDC2 conferred higher risk in COAD and PRAD (Supplementary Figures S4D,E). PFI analysis identified favorable associations in BLCA, HNSC, KIRC, KIRP, LUAD, MESO and PAAD, but an adverse impact in LGG and PRAD (Supplementary Figures S5A–S).

Pan-cancer immune deconvolution showed positive correlations between ECHDC2 and B cells, follicular helper T (TFH) cells and natural-killer (NK) cells, and negative correlations with macrophages, myeloid-derived suppressor cells (MDSC) and cancer-associated fibroblasts (CAF) (Supplementary Figure S6A).

## 4 Discussion

Despite substantial progress in the diagnosis and treatment of GBM, patient prognosis remains bleak (Stupp et al., 2005; Wen and Kesari, 2008; Ostrom et al., 2019; Jiang et al., 2022). The discovery of reliable biomarkers is therefore crucial for refining personalized therapeutic strategies. In the present study, we demonstrated that high ECHDC2 expression correlates with multiple indicators of aggressive disease—namely, advanced WHO grade, unmethylated MGMT promoter, IDH-wild-type status, 1p/19q non-codeletion and unfavorable histology. Consistently, patients with elevated tumoral ECHDC2 exhibited shortened OS and PFS, underscoring ECHDC2 as a tumor-promoting factor in GBM.

Notably, single-cell transcriptome analysis revealed that ECHDC2 is expressed not only in malignant cells, but also in Astrocyte cells, T cells and myeloid cells (Figures 2A,B), suggesting that it may exert compartment-specific functions (Gajewski et al., 2013; Propper and Balkwill, 2022). Although the present study focuses on elucidating the pro-tumorigenic role of ECHDC2 within tumor cells, its expression in immune cells may be closely associated with the immune regulation of the tumor microenvironment. For instance, the positive correlation between high ECHDC2 expression and immune-related molecules such as LIF and TNFSF14/TNFRSF14 (Rose-John, 2018; Pinho et al., 2020; Loriot et al., 2021; Jorgensen and de la Puente, 2022), together with its association with the infiltration of TEM and pDCs, implies that ECHDC2 may indirectly facilitate tumor progression by regulating immune cell functions or intercellular communication (Pascual-García et al., 2019; Peñuelas et al., 2009) (Figures 4A–J). Future studies should employ conditional knockout, cell-type-specific knockdown or co-culture systems to further dissect the distinct functions of ECHDC2 in tumor cells versus immune/stromal cells, as well as their reciprocal interactions.

The present study found that high ECHDC2 expression was positively correlated with the infiltration levels of TEM and pDCs in the tumor microenvironment (Figures 4D,I,J). TEM typically represent anti-tumor effector cells with robust reactivation potential, and their presence is often associated with favorable prognosis in “cold tumors” such as GBM (Tang et al., 2026). However, the immunosuppressive microenvironment of GBM can induce functional exhaustion or dysfunction of these cells (Eoli et al., 2019; Kurachi, 2019). pDCs are a specialized subset of dendritic cells that generally induce immune activation via the production of type I interferons in tumors; yet in the context of chronic inflammation or malignancy, they are more frequently reported to promote immune tolerance and poor prognosis by inducing regulatory T cells and expressing immunosuppressive molecules (Balan et al., 2019; Li S. et al., 2017; Faget et al., 2013; Xiao et al., 2021). Therefore, the enrichment of TEM and pDCs accompanying high ECHDC2 expression may not equate to effective anti-tumor immune activation, but rather may reflect a state of immune dysregulation or dysfunction. We found that high ECHDC2 expression was significantly correlated with the expression of LIF and TNFSF14/TNFRSF14 (Figures 4A,B,E–G). LIF has been demonstrated to inhibit CD8^+^ T cell tumor infiltration and recruit immunosuppressive macrophages (Pascual-García et al., 2019; Peñuelas et al., 2009). TNFSF14 signaling exhibits complex dual roles in tumors, potentially affecting both angiogenesis and immune cell function simultaneously (Ramachandran et al., 2023; Zottel et al., 2025; Yang et al., 2021; Han et al., 2022). ECHDC2 may create an immune microenvironment characterized by apparent immune cell infiltration but impaired function through upregulating these molecules. Collectively, the pro-tumorigenic effect of ECHDC2 is a multi-faceted process: its cell-intrinsic functions in tumor cells drive proliferation and invasion; meanwhile, the specific immune cell infiltration pattern associated with its expression may collectively contribute to a microenvironment that favors tumor growth rather than immune elimination. This explains why high ECHDC2 expression generally predicts poorer clinical outcomes.

Fatty-acid β-oxidation (FAO) supplies ATP and biosynthetic precursors and supports GBM growth (Son et al., 2020; Wang et al., 2019). Members of the enoyl-CoA hydratase/isomerase family are indispensable for FAO (Padavattan et al., 2021; Müller-Newen et al., 1995). We found that high expression of ECHDC2 was correlated with poor clinical prognosis, whereas ECHDC2 knockdown impaired the proliferation, migration, and invasion abilities of GBM cells *in vitro*. Aberrant activation of the PI3K/Akt pathway plays a pivotal role in GBM progression (Braccini et al., 2012) and also regulates metabolic reprogramming in GBM cells (Guo et al., 2009; Singh et al., 2025). Consistent results from bioinformatics analyses indicated that ECHDC2 is associated with the PI3K/Akt pathway. Specifically, the level of p-AKT was significantly decreased in ECHDC2-knockdown cells, while it was increased in ECHDC2-overexpressing cells, with no obvious alterations in total AKT expression (Figures 5F,G). These findings support a model wherein ECHDC2 maintains the aggressive phenotype of GBM by upregulating the PI3K/Akt pathway. However, the mechanistic exploration in the present study remains preliminary. Further investigations using immunoprecipitation-mass spectrometry (IP-MS), co-immunoprecipitation (Co-IP), and other relevant assays should be performed to clarify the specific molecular mechanisms underlying ECHDC2-mediated regulation of the PI3K/Akt pathway.

Pan-cancer analyses extended ECHDC2’s relevance beyond GBM. High expression associated with OS, DSS, DFI or PFI in several tumor types and correlated broadly with immune-cell infiltration, including TFH cells—which orchestrate tertiary lymphoid structures (Song and Craft, 2024)—and tumor-associated macrophages (TAMs) (Belgiovine et al., 2016; Shapouri‐Moghaddam et al., 2018). Thus, ECHDC2 may act as a context-dependent regulator of tumor immunity and metabolism across cancers.

Our study has certain limitations. Public database cohorts are susceptible to batch effects and sampling bias. The mechanistic links between ECHDC2, immune infiltration, and patient prognosis remain purely correlative rather than causal. The exact druggability of ECHDC2 remains to be verified. Future studies need to employ high-throughput drug screening or structure-based drug design to identify and validate compounds that can specifically target ECHDC2 or its functional pathways. Finally, functional validation has been limited to *in vitro* experiments; comprehensive *in vivo* studies are required to elucidate the molecular programs of ECHDC2 in GBM.

## 5 Conclusion

ECHDC2 emerges as a clinically relevant biomarker of poor prognosis in GBM. Its high expression associates with aggressive clinicopathological features and enriched immune-cell infiltration. Functional inhibition of ECHDC2 can suppress the proliferation, migration, and invasion of GBM cells, which provides robust preliminary evidence and a theoretical rationale for further exploring ECHDC2 as a prospective therapeutic target for GBM in future research.

## Data Availability

The datasets presented in this study are available in the Gene Expression Omnibus repository under the accession numbers GSE43378, GSE13041, GSE74187, GSE83300, and GSE182109, as well as in The Cancer Genome Atlas and the Chinese Glioma Genome Atlas repositories.
