# Supplementary material for: Clinical significance and oncogenic role of ECHDC2 in glioblastoma: a comprehensive analysis based on bioinformatics and in vitro experiments
Source: Front Genet. 2026 Feb 9;17:1759463. doi: 10.3389/fgene.2026.1759463 (PMC12925631; doi:10.3389/fgene.2026.1759463)
Supplement: Supplementary file 1 [file DataSheet4.docx]

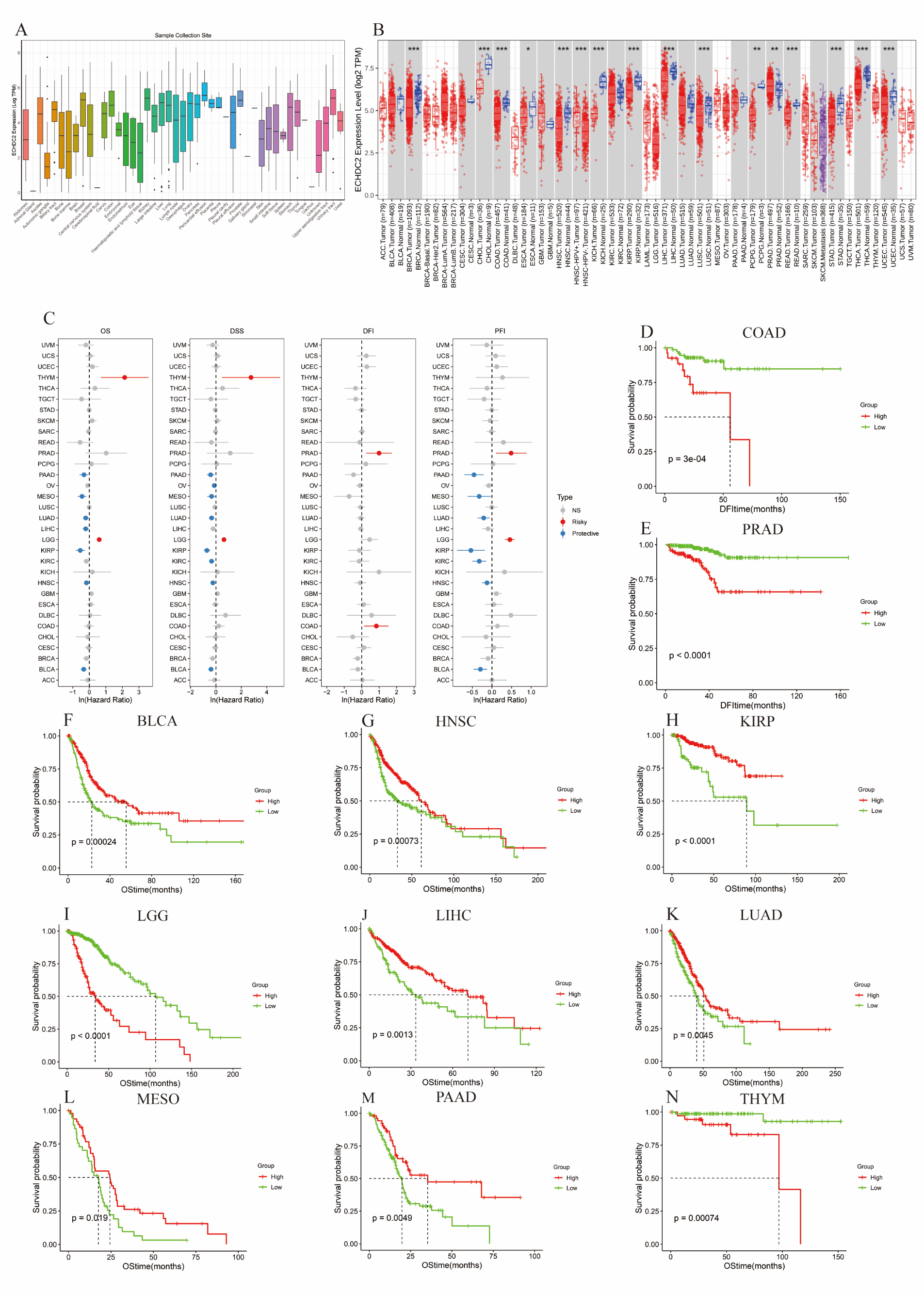


**Supplemental figure 4. Pan-cancer expression landscape of ECHDC2 and its prognostic significance.**
(A) Boxplots depicting ECHDC2 mRNA levels across normal human tissues profiled in the CCLE dataset. (B) Boxplots comparing ECHDC2 expression in tumour tissues versus matched normal tissues across cancer types in the TIMER database. (C) Forest plot summarising univariate Cox regression analyses of ECHDC2 expression for OS, DSS, DFI and PFI across TCGA cancer types. (D, E) Kaplan–Meier curves illustrating DFI stratified by high- versus low-ECHDC2 expression in representative cancer types. (F–N) Kaplan–Meier curves of OS for high- and low-ECHDC2 expression groups across additional cancer types. Abbreviations: OS, overall survival; DSS, disease-specific survival; DFI, disease-free interval; PFI, progression-free interval; CCLE, Cancer Cell Line Encyclopedia; TIMER, Tumour Immune Estimation Resource; TCGA, The Cancer Genome Atlas.
