# Supplementary material for: Clinical significance and oncogenic role of ECHDC2 in glioblastoma: a comprehensive analysis based on bioinformatics and in vitro experiments
Source: Front Genet. 2026 Feb 9;17:1759463. doi: 10.3389/fgene.2026.1759463 (PMC12925631; doi:10.3389/fgene.2026.1759463)
Supplement: Supplementary file 2 [file DataSheet6.docx]

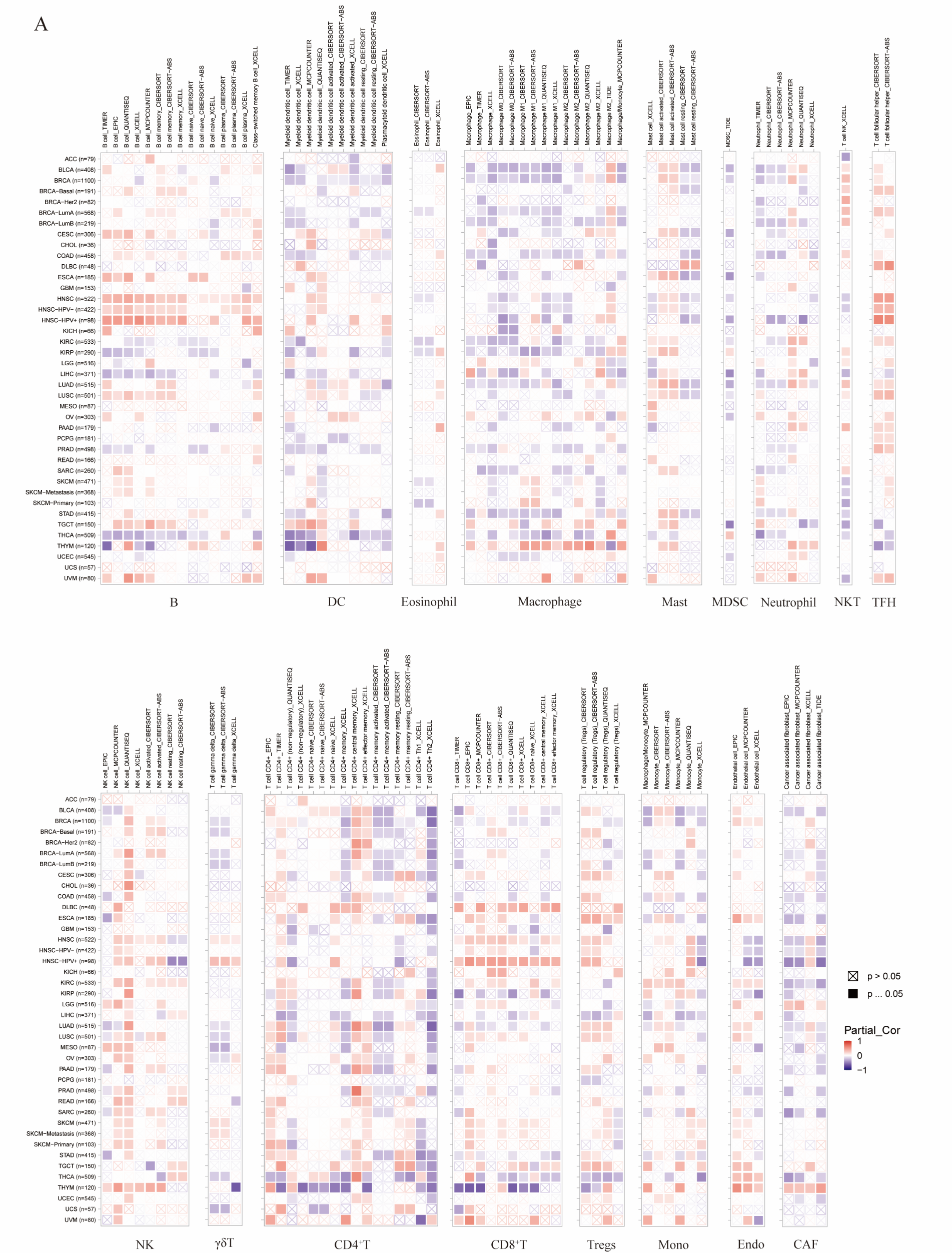


**Supplementary Figure 6. Correlation between ECHDC2 expression and immune-cell infiltration across cancers.**
(A) Clustered heat-map showing the correlation coefficients between ECHDC2 mRNA abundance and the estimated infiltration levels of B cells, dendritic cells (DCs), eosinophils, macrophages, mast cells, MDSCs, neutrophils, natural-killer T (NKT) cells, TFH, NK cells, γδ T cells, CD4+ T cells, CD8+ T cells, regulatory T cells (Treg), monocytes, endothelial cells and CAF, as computed with the TIMER online platform. Red squares indicate positive correlations, blue squares indicate negative correlations, squares marked with a cross denote non-significant correlations (P > 0.05) and black-bordered squares denote statistically significant correlations (P < 0.05).
