# Supplementary material for: Clinical significance and oncogenic role of ECHDC2 in glioblastoma: a comprehensive analysis based on bioinformatics and in vitro experiments
Source: Front Genet. 2026 Feb 9;17:1759463. doi: 10.3389/fgene.2026.1759463 (PMC12925631; doi:10.3389/fgene.2026.1759463)
Supplement: Supplementary file 3 [file DataSheet3.docx]

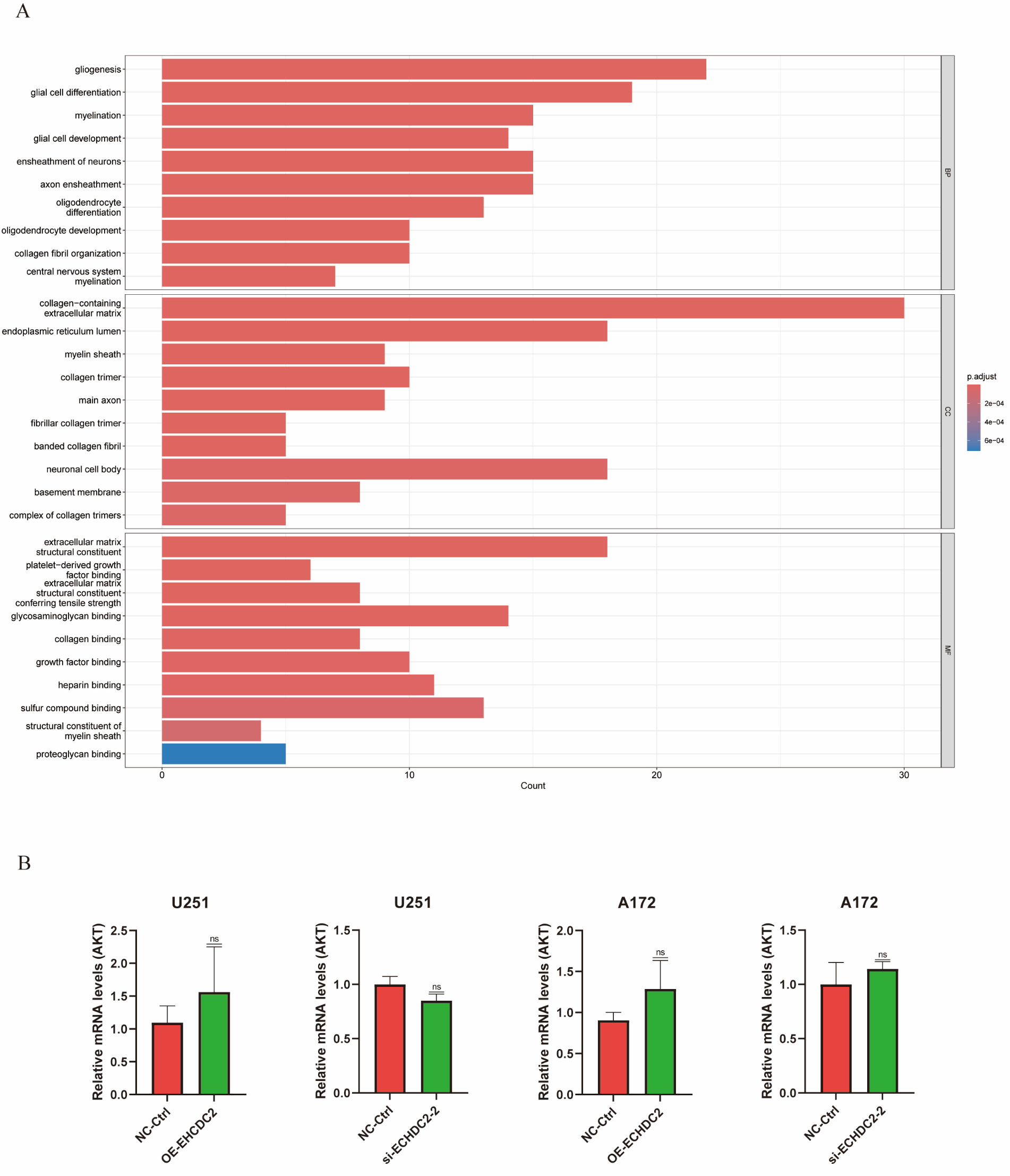


**Supplementary Figure 3. GO enrichment analyses and impact of ECHDC2 on the PI3K/Akt signaling pathway.**

(A) GO enrichment analyses for the ECHDC2-high and -low groups in CGGA-325 cohort. (B) qPCR analyses of AKT in GBM cells with ECHDC2 overexpression or knockdown.
