# Supplementary material for: Clinical significance and oncogenic role of ECHDC2 in glioblastoma: a comprehensive analysis based on bioinformatics and in vitro experiments
Source: Front Genet. 2026 Feb 9;17:1759463. doi: 10.3389/fgene.2026.1759463 (PMC12925631; doi:10.3389/fgene.2026.1759463)
Supplement: Supplementary file 4 [file DataSheet5.docx]

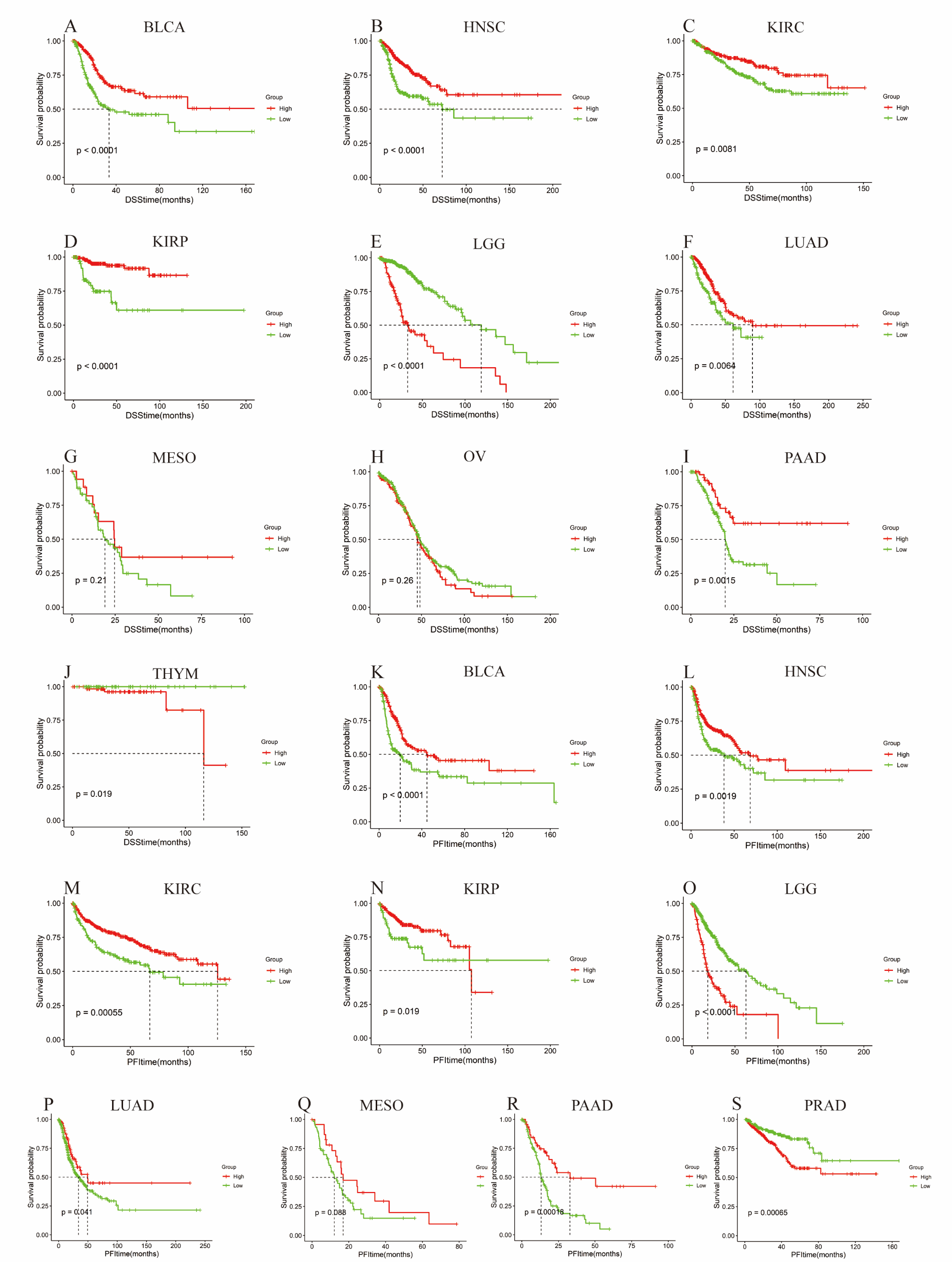


**Supplementary Figure 5. Prognostic impact of ECHDC2 expression across human cancers.**
(A–J) Kaplan–Meier curves depicting DSS in cohorts stratified by high- versus low-ECHDC2 expression across multiple tumor types. (K–S) Kaplan–Meier curves illustrating PFI in cohorts stratified by high- versus low-ECHDC2 expression across tumor types. Abbreviations: DSS, disease-specific survival; PFI, progression-free interval.
