# Supplementary material for: Clinical significance and oncogenic role of ECHDC2 in glioblastoma: a comprehensive analysis based on bioinformatics and in vitro experiments
Source: Front Genet. 2026 Feb 9;17:1759463. doi: 10.3389/fgene.2026.1759463 (PMC12925631; doi:10.3389/fgene.2026.1759463)
Supplement: Supplementary file 5 [file DataSheet2.docx]

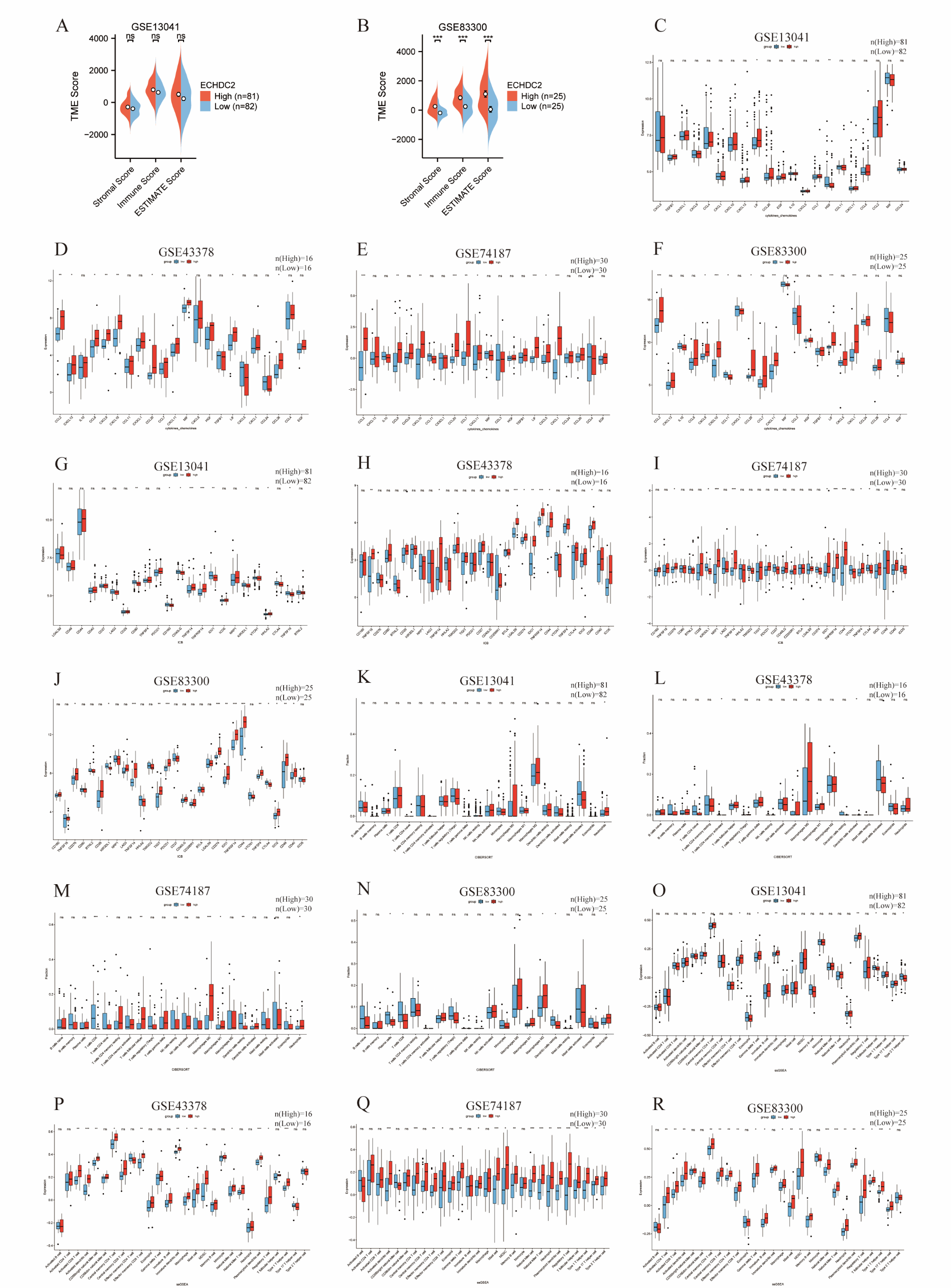


**Supplementary Figure 2. Immune-infiltration landscape associated with ECHDC2 expression in GBM.**
(A-B) Stromal, Immune, and ESTIMATE Scores were compared between the high- and low-ECHDC2 expression groups, as calculated by the ESTIMATE algorithm, in the GSE13041 and GSE83300 cohorts. (C-F) Expression levels of cytokine and chemokine genes were compared between ECHDC2-high and -low groups across the GSE13041, GSE43378, GSE74187, and GSE83300 cohorts. (G-J) Differential expression of immune checkpoint blockade (ICB) genes between ECHDC2-high and -low groups across the GSE13041, GSE43378, GSE74187, and GSE83300 cohorts. (K-R) The relative abundance of tumor-infiltrating immune cell subsets in ECHDC2-high versus -low tumors was assessed by CIBERSORT and ssGSEA analyses in the GSE13041, GSE43378, GSE74187, and GSE83300 cohorts. Significance: ns: no significance; *P < 0.05; **P < 0.01; ***P < 0.001; ****P < 0.0001.
