# Supplementary figures and images for: Clinical significance and oncogenic role of ECHDC2 in glioblastoma: a comprehensive analysis based on bioinformatics and in vitro experiments
Source: Front Genet. 2026 Feb 9;17:1759463. doi: 10.3389/fgene.2026.1759463 (PMC12925631; doi:10.3389/fgene.2026.1759463)

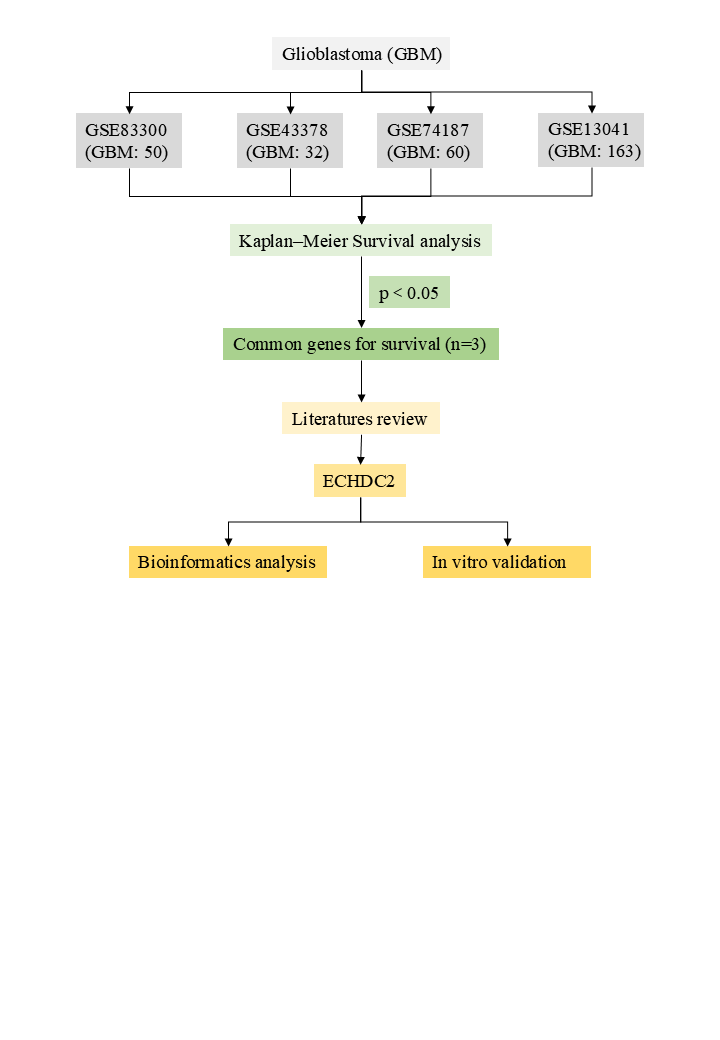


**Supplementary Figure 1** Flowchart of study design. GBM: glioblastoma.

Supplement: Supplementary file 7 [file DataSheet1.docx]
